# A DETROIT-BASED COALITION TO PROMOTE EQUITY AT THE INTERSECTION OF HEALTH, HOUSING, AND AGING

**DOI:** 10.1093/geroni/igae098.1963

**Published:** 2024-12-31

**Authors:** Tam Perry, Melissa Draughn, Claudia Sanford, Dennis Archambault, Zach Kilgore, Michele Watkins

**Affiliations:** Wayne State University, Detroit, Michigan, United States; Hannan Center, Detroit, Michigan, United States; United Community Housing Coalition, Detroit, Michigan, United States; Authority Health, Detroit, Michigan, United States; CSI Support & Development, Warren, Michigan, United States; Volunteers of America Michigan, Southfield, Michigan, United States

## Abstract

This presentation focuses on a multi-sector coalition focused on senior housing. Since its founding in 2013, Senior Housing Preservation-Detroit has worked tirelessly to address the displacement of older adults, the loss of senior housing as the city gentrifies; and ways to improve quality of life to those living in senior housing. This presentation will focus on the decade long work achieved respect among Detroit elected officials and housing staff through direct advocacy. Members have also testified at Detroit City Council meetings and participated in numerous local, state, national, and international forums on affordable housing for older adults. To date, coalition members have published four peer-reviewed articles; three invited articles; one white paper; and two book chapters as well as a documentary. The work of the coalition also involves numerous agencies serving older adults in Detroit. The Special Commission to Advance Macro Social Work awarded the coalition the Jack Rothman award for Structural Change in 2023. The presentation will focus on milestones, lessons learned, and the coalition’s COVID-19 outreach in senior buildings. It will offer perspectives on its current initiative on preparing an emergency preparedness plan for Detroit’s older population.

